# Validity of the International Physical Activity Questionnaire (short form) in adults with asthma

**DOI:** 10.1371/journal.pone.0282137

**Published:** 2023-02-24

**Authors:** Joice Mara Oliveira, Thamyres Spositon, Diery Fernandes Rugila, Fabio Pitta, Karina Couto Furlanetto

**Affiliations:** 1 Research and Postgraduate Center, Pitágoras-Unopar University (UNOPAR), Londrina, Paraná, Brazil; 2 Laboratory of Research in Respiratory Physiotherapy (LFIP), State University of Londrina, Londrina, Paraná, Brazil; University of Pavia: Universita degli Studi di Pavia, ITALY

## Abstract

**Background:**

The short form of the International Physical Activity Questionnaire (IPAQ) is widely used to assess PA and has already been used in adults with asthma; however, its validity has not been yet studied in this population. Therefore, the aim of this study was to verify the convergent and discriminative validity of the IPAQ short form in adults with asthma.

**Methods:**

Fifty-three adults with asthma (36 females; 48±15 years; 29±6 kg/m²) wore the triaxial activity monitor Actigraph for eight days to objectively measure steps/day, time in light physical activity (PA), moderate-to-vigorous PA (MVPA), and sedentary behaviour. Participants filled out the IPAQ matching with the same week they wore the Actigraph, with measures of: time of MVPA and total PA/week; categorization of low, moderate or high PA level; time in seated position.

**Results:**

IPAQ self-reported total time of PA/week was weakly correlated with steps/day. The IPAQ categorization correlated moderately with time in light, MVPA and steps/day. Self-reported time in seated position on weekdays was moderately correlated with objective percentage/day of time in sedentary behaviour in the same period. IPAQ categorization in PA levels was able to differentiate between low to moderate and low to high PA levels.

**Conclusions:**

These results cannot confidently infer the convergent validity of the IPAQ to quantify number of steps/day and time spent in PA of adults with asthma. However, this instrument may be useful to categorize patients into three levels of PA.

## Introduction

Physical inactivity and sedentary behaviour have been considered a worldwide problem, since they are related to all-cause mortality, incidence of cardiovascular disease, type 2 diabetes, cancer, and other outcomes [1]; nevertheless, 27.5% of the general adult population do not meet the international recommendations [2]. This percentage is even greater considering people with asthma, who undertake less physical activity (PA) than people without the disease [3]. Physical activity has been increasingly investigated in this population, and it is already known that adults with asthma who are physically inactive have higher risk of asthma symptoms, poorer asthma control and health status [4]. Therefore, assessment of PA is an important outcome in people with asthma.

There are different ways to assess PA, either objectively or subjectively [5]. One of the most used objective methods is accelerometry, which does concurrent measure of movements for weeks and provides data on PA duration, frequency and intensity [5, 6]. However, accelerometers are generally used on the hip, thus eventually neglecting upper-limb activities. They may also not measure properly activities that require lifting a load, climbing stairs or cycling [5]. Lastly, accelerometers can be relatively expensive when compared to subjective measures, such as questionnaires or diaries [5, 7].

Questionnaires which assess PA may overcome, at least partially, some of the limitations inherent to accelerometry. Despite presenting limitations, such as recall bias and low validity for assessing casual or lifestyle PA [5, 8, 9], questionnaires are an easy-to-use and low-cost tool, therefore allowing application in large populations. In addition, questionnaires can assess different PA dimensions and domains, can be done in a single time point and cause low burden to participants [5, 7], although they need to be population and culture specifically validated [5].

The International Physical Activity Questionnaire (IPAQ) is widely used to assess PA [8, 10] both on its long or short forms. Its long form contains 31 items divided in 6 domains (occupational, transport, yard, household, leisure and sitting). The IPAQ short form (8 items) encompasses questions regarding time spent walking, in moderate and vigorous intensity PA, besides sitting time [10]. The short form might be preferred because it is feasible to administer and shows similar reliability and validity results in comparison to the long form [10]. The IPAQ short form has already been used in other studies involving adults with asthma [4, 11, 12], however, its validity has not been yet established in this population.

Verifying psychometric properties such as validity is important to check the degree to which an instrument truly measures the construct it purports to measure [13]. Construct validity is usually divided in specific types, and a quite useful type is the concurrent validity, which measures the correlation between scores of the instrument of study and a measure of some other instrument in which a convergent construct is expected (in the absence of a gold standard). It should also be checked whether the instrument can discriminate different groups (discriminative validity–also known as “known groups”) [13].

Several studies have analysed the IPAQ short form validity and showed that it may overestimate PA, in addition to the fact that its correlations with PA or fitness measures are low [8]. Therefore, it is essential to certify if this instrument is valid before using it in a given population, e.g., adults with asthma. Thus, the aim of this study was to verify the convergent and discriminative validity of the IPAQ (short form), as well as its agreement with objectively measured PA in adults with asthma. We hypothesized that the IPAQ short form would have low-moderate correlations with the objectively measured PA (convergent validity) and that this questionnaire would be able discriminate different PA levels in this population.

## Methods

We conducted this cross-sectional study that included adults with diagnosis of asthma according to the Global Strategy for Asthma Management and Prevention (GINA) [14], under medical treatment for at least 6 months; with clinical stability (i.e., no exacerbation symptoms or increment in asthma medication for at least 30 days), and without concomitant limiting cardiovascular and/or musculoskeletal diseases. Exclusion criteria were exacerbation or changes in asthma medication during the assessment period, diagnosis of other pulmonary disease(s) and to decline finalizing the assessments for any reason. Patients from the University Hospital of Londrina (Brazil) Pulmonology outpatient clinic and others who sought us out responding to advertisements were invited to participate in the study. Data collection was performed between April 2018 and June 2019. The study was approved by Pitagoras-Unopar University Ethics Committee (number 3.060.314) and all participants signed and received an informed consent copy.

All participants were assessed regarding sociodemographic (sex and age) and anthropometric data (objectively measured height and weight followed by calculation of the body mass index). Lung function was accessed using a spirometer (MicroLab 3500, Care Fusion®, Ireland) in order to measure forced expiratory volume in the first second (FEV_1_) and forced vital capacity (FVC) according to international guidelines [15]. Reference values for the Brazilian population were used [16]. Asthma control was assessed through the Asthma Control Questionnaire (ACQ) [17].

Subjects also received a triaxial activity monitor Actigraph wGT3X-BT with instructions to wear the device on their waist aligned with the right hip, during the whole day for 8 consecutive days (removing it only for showering and occasional water activities). The device was initialized using Actilife 6 with 60-s epoch [18]; data were downloaded and analysed in the same software. Sleep period data were deleted and the minimum wearing criteria were ≥4 days and ≥10 hours of wearing time each day [19, 20]. Main outcome variables were time spent per day in light PA (100–1951 counts per minute–cpm), moderate-to-vigorous PA (MVPA; ≥1952 cpm), and sedentary behaviour (<100 cpm) [18] in weekdays and in weekend days, as well as the average number of steps/day, as these are the most commonly used measures of PA [21].

The IPAQ-short form consists of 8 questions with recall period defined as ‘last week’ and was answered immediately at the end of the Actigraph PA assessment week. The questions of the instrument ask whether the individual walked or performed moderate or vigorous activities for at least 10 minutes on any day, and how much time/day they spent on each of these activities. The last questions ask about the time that the individual remains seated during a weekday and a weekend day. IPAQ variables analysed were: time spent in MVPA and total PA (min/week); the categorization as low, moderate or high PA level (according to frequency and duration of PA and METs/week); and the time spent in the seated position [8].

The Statistical Package for Social Sciences (SPSS) version 22 (SPSS Inc, Chicago, USA) was used. Normality of the data distribution was verified by the Shapiro-Wilk test. To test the convergent validity, the Spearman Correlation Coefficient was used to verify correlations between Actigraph and IPAQ data (reported as “rs” values), as the latter presented non-normally distributed data. One-way ANOVA with Tukey post-hoc, or Kruskal-Wallis with Dunn’s post-hoc were used, according to data distribution, to test the discriminative validity for the IPAQ categorization in low, moderate or high PA levels. Differences between men and women were analysed through independent T-student test or Mann-Whitney test, according to data distribution. Agreement between IPAQ and Actigraph data was verified by plotting their difference against means (Bland-Altman plots) [22]. Statistical significance was set at P<0.05. This study met the Cosmin (COnsensus‐based Standards for the selection of health Measurement Instruments) [23] standards of study design and statistical analysis for validation studies. The sample was derived from a previous unrelated study [24], and its size also met the Cosmin recommendations (minimum of 50) [23, 25].

## Results

Fifty-three participants were analysed. The majority of the sample was middle-aged, overweight and presented partially controlled to controlled asthma. According to GINA, 72% of the sample had severe asthma and 28% had mild-moderate asthma. Characteristics of the sample are shown in Table 1. There were no differences between men and women in any variables used in this study (P ≥ 0.09 for all).

**Table 1:** Characteristics of the sample and results of physical activity assessed by both instruments (Actigraph and IPAQ-short form).

	N = 53
**Sex (men/woman)**	17/36
**Age (years)**	48±15
**Body Mass Index (kg/m²)**	29±6
**FEV**_**1**_ **(L)**	2.19±0.71
**FEV**_**1**_ **(%predicted)**	71±16
**FEV**_**1**_ **/FVC (%)**	70±11
**Asthma Control (ACT score, 5–25)**	19±4
**Physical activity, Actigraph**	
**Light physical activity (minutes/day)**	350±89
**Light physical activity (minutes/week)**	2632±760
**MVPA (minutes/day)**	24±21
**MVPA (minutes/week)**	184±162
**Steps (mean/day)**	7078±3179
**Steps (mean/week)**	53305±25608
**Sedentary weekday (minutes/day)**	551±95
**Sedentary weekday (% of the day)**	59±10
**Sedentary weekend (minutes/day)**	546±106
**Sedentary weekend (% of the day)**	61±10
**Physical activity, IPAQ short form**	
**MVPA (minutes/week)**	597±622
**Total PA (minutes/week)**	831±915
**IPAQ categorization, low/moderate/high PA (n, %)**	16 (30)/18(34)/19(36)
**Seated position weekday (minutes)**	301±185
**Seated position weekend day (minutes)**	299±198

Data are presented as absolute value, relative value or mean±SD. FEV_1_: forced expiratory volume in the first second; FVC: forced vital capacity; ACT: Asthma Control Test; MVPA: moderate-vigorous physical activity; PA: physical activity; IPAQ: International Physical Activity Questionnaire.

Regarding PA assessed by the IPAQ, time spent in MVPA was not significantly correlated with objectively measured MVPA (rs = 0.19; P = 0,169). The IPAQ self-reported total time of PA/week was weakly correlated with time spent in light PA (rs = 0.26; P = 0.058), MVPA (rs = 0.27; P = 0.050) and steps/day (rs = 0.32; P = 0.020) assessed by the Actigraph. On the other hand, the IPAQ categorization correlated moderately with time spent in light PA, MVPA and steps/day (rs = 0.44, 0.41, and 0.51, respectively; P≤0.003 for all). In addition, IPAQ’s self-reported time spent in the seated position on weekdays was moderately correlated with the Actigraph’s percentage/day of time spent in sedentary behaviour in the same period (rs = 0.41; P = 0.003). All other correlations were not statistically significant and/or clinically irrelevant. Moreover, 70% of the sample self-reported performing more time in MVPA than objectively measured by the Actigraph.

Comparisons of objectively measured PA data between the IPAQ categorization in low, moderate or high PA levels are shown in Table 2. These results show that there were differences in PA measured by the Actigraph between the 3 categories of IPAQ. However, in the post-hoc analysis the differences between the IPAQ moderate and high PA levels were not statistically significant.

**Table 2:** Comparisons of objectively measured physical activity variables (assessed by the Actigraph) among the three categories of the International Physical Activity Questionnaire (IPAQ)-short form.

	IPAQ low PA	IPAQ moderate PA	IPAQ high PA	P
**Light PA (minutes/week)**	2123±413 *#	2776±763	2924±797	0.003
**MVPA (minutes/week)**	94±79 #	186±125	260±207	0.008
**Steps/week**	34908±10564 *#	59216±24759	63198±27988	<0.0001

Data are presented as mean±SD. MVPA: moderate-vigorous physical activity; PA: physical activity; *post-hoc P<0.05 *vs* IPAQ moderate PA; # post-hoc P<0.05 *vs* IPAQ high PA.

**Fig 1:**
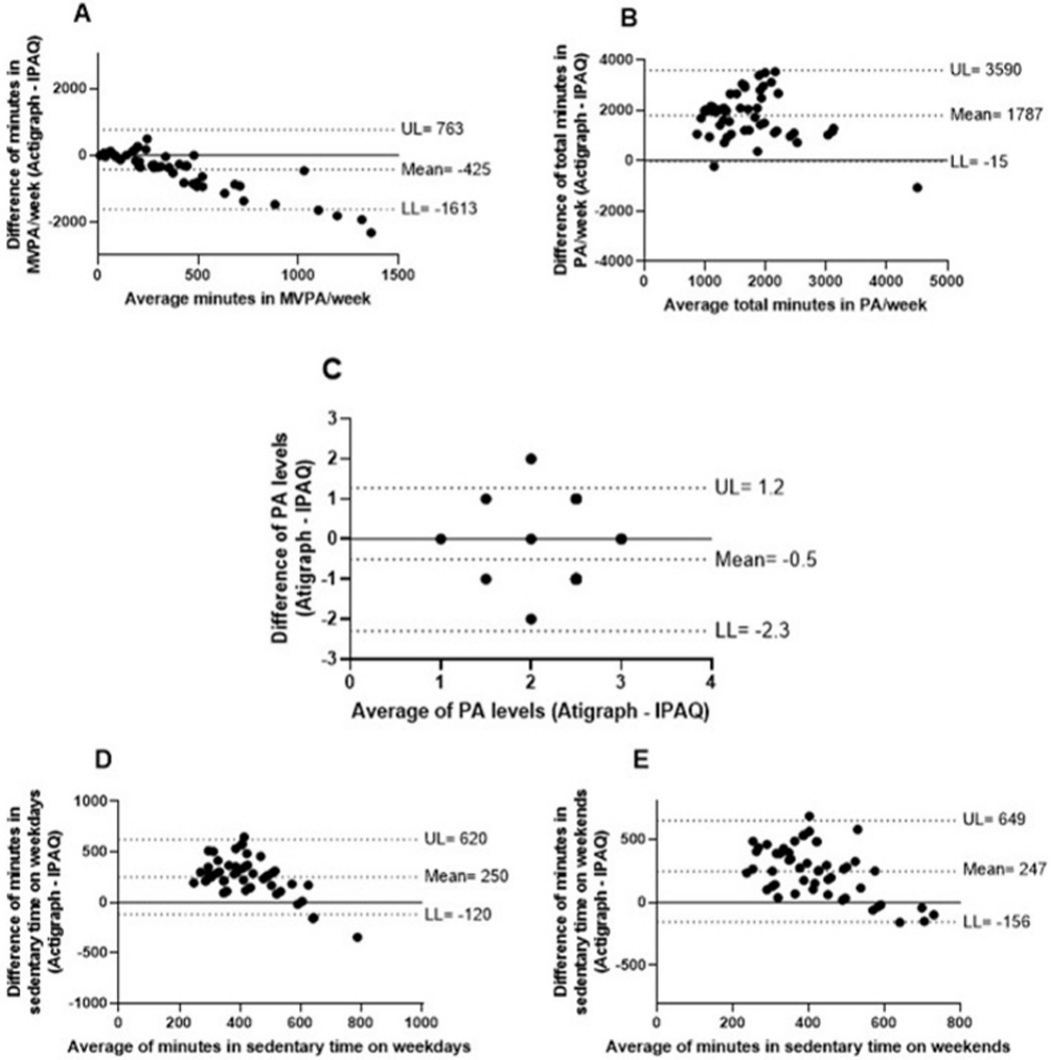
Bland-Altman plots for agreement between the International Physical Activity Questionnaire (IPAQ)-short form and measured physical activity (Actigraph) in the variables: moderate-to-vigorous physical activity (MVPA) per week (A); total minutes in PA per week (B); categorization in PA levels (C); minutes in sedentary time on weekdays (D) and on weekends (E).

Agreement between IPAQ and Actigraph data can be seen in the Bland-Altman plots (Fig 1). In plot A, IPAQ seems to better agree with Actigraph in patients who had short MVPA time, and tends to overestimate MVPA as time increases. On the other hand, total minutes in PA/week (plot B), minutes in sedentary time on weekdays (plot D) and on weekends (plot E) had their means far from zero, with a tendency for IPAQ to underestimate the Actigraph values. Lastly, the PA levels (plot C) had a mean closer to zero and a distribution within 95% confidence interval.

## Discussion

The present study showed very modest or clinically irrelevant correlations between time of MVPA or total PA/week reported with the IPAQ and the variables objectively measured by the Actigraph. On the other hand, IPAQ categorization presented better correlation coefficients with objective measurements than using IPAQ variables reported in absolute values. Additionally, IPAQ categorization in PA levels was able to differentiate objectively measured PA, especially low from moderate and low from high levels. Although there is no statistical difference between the moderate and high IPAQ levels of PA in the post-hoc analysis, the mean values of light PA, MVPA and steps per week (measured by the Actigraph) increase according to each IPAQ PA categorization level. This is in accordance with previous studies in other populations [8].

Accordingly, the Bland-Altman plots did not appear to have good agreement between IPAQ and the objectively measured PA, except for the categorization in PA levels, which showed better results. By using the questionnaire, adults with asthma overestimated their time spent in MVPA, as well as reported less time in total PA and in the seated position. Of note, there was moderate correlation between the latter variables in the weekdays; these results are similar to those found on a recent study in older adults without the disease [26].

Regarding MVPA overestimation, this might happen due to the physiopathology of asthma, which leads these subjects to feel more breathlessness during physical activities (i.e., sensation of higher intensity) than those without the disease [27]. This way, by answering the questionnaires people with asthma report more time in MVPA because they might really feel that they are doing a more strenuous effort during activities. On the contrary, the activity monitor (Actigraph) assesses what they actually do, measured through movement and acceleration, without taking into account their sensations. Previous studies in other populations have also shown that IPAQ-short form tends to overestimate the amount of PA compared to an objective assessment tool [8].

Ideally, validation studies should have a larger sample; however, we have met the Cosmin minimum recommendations (N = 50) [23, 25]. Another limitation of this study is to have different proportions of asthma severity. On the other hand, we included in the sample all asthma severities and a large age range (from 19 to 81 years old), which can contribute to the external validity and generalizability of the present results. Furthermore, the strength of this study is to have performed the validation analysis against an objective assessment (accelerometer), which is not done in all validity studies.

## Conclusions

The present results cannot confidently infer adequate convergent validity and agreement of the IPAQ-short form to estimate number of steps/day and time spent in PA of adults with asthma. However, the instrument reflects moderately the time spent in sedentary behaviour. Furthermore, despite its usefulness in the categorization of low levels of PA, the IPAQ-short form was not able to statistically discriminate between moderate and high levels of PA in the present sample.

## Supporting information

S1 Dataset Data underlying the findings. (XLSX)
